# Editorial: Precision medicine approaches for heterogeneous conditions such as autism spectrum disorders (The need for a biomarker exploration phase in clinical trials - Phase 2m)

**DOI:** 10.3389/fpsyt.2022.1079006

**Published:** 2023-01-19

**Authors:** David Q. Beversdorf, Evdokia Anagnostou, Antonio Hardan, Paul Wang, Craig A. Erickson, Thomas W. Frazier, Jeremy Veenstra-VanderWeele

**Affiliations:** ^1^Departments of Radiology, Neurology, and Psychological Sciences, William and Nancy Thompson Endowed Chair in Radiology, University of Missouri, Columbia, MO, United States; ^2^Holland Bloorview Kids Rehabilitation Hospital, University of Toronto, Toronto, ON, Canada; ^3^Division of Child and Adolescent Psychiatry, Department of Psychiatry and Behavioral Sciences, Stanford University, Stanford, CA, United States; ^4^Clinical Research Associates LLC, Simons Foundation, Department of Pediatrics, Yale University School of Medicine, New Haven, CT, United States; ^5^Division of Child and Adolescent Psychiatry, Cincinnati Children's Hospital Medical Center, Cincinnati, OH, United States; ^6^Department of Psychiatry and Behavioral Neuroscience, University of Cincinnati College of Medicine, Cincinnati, OH, United States; ^7^Department of Psychology, John Carroll University, University Heights, OH, United States; ^8^Department of Pediatrics, State University of New York Upstate Medical University, Syracuse, NY, United States; ^9^Departments of Psychiatry and Pediatrics, New York State Psychiatric Institute, Columbia University, New York, NY, United States; ^10^NewYork-Presbyterian Center for Autism and the Developing Brain, New York, NY, United States

**Keywords:** autism (ASD), heterogeneity, treatment, genetics, biomarkers

Significant progress has been made in understanding the biology of autism spectrum disorder (ASD), providing rational hypotheses for interventions to address the core symptoms. However, clinical trials of these interventions have failed to yield positive results to date. In many of these studies, a subset of participants appear to respond well, but a significant benefit is not found in the overall intent-to-treat group. Due to the etiological heterogeneity of ASD, we anticipate that this will continue to be a challenge in future clinical trials. It will be critical to identify the patients that are most likely to respond to a treatment and to target those subjects in later phase trials. We therefore propose the explicit inclusion of “Phase 2m” as part of the pathway of clinical drug development, specifically for the development of a biomarker profile that can be incorporated into later phase 2 and 3 clinical trials. Such a precision medicine approach has the potential to optimize the likelihood of success in future clinical trials to benefit patients.

## Introduction

Autism spectrum disorder (ASD) is highly heterogeneous, with estimates of potentially 1,000 genes that may be associated with risk for ASD (1). This is in addition to cases with environmental and other non-genetic contributors such as infection and inflammation during pregnancy (2), as well as cases that would at this point be considered idiopathic cases. Studies using cellular and animal models have pointed to underlying neurobiological systems and pathways impacted by individual ASD risk genes, with some suggestion of convergence across genes. For example, research exploring the synaptic mechanisms impacted by the fragile X syndrome (FXS) gene *FMR1* led to trials with negative allosteric modulators of metabotropic glutamate type 5 receptors in FXS (3). Converging lines of evidence (4, 5) led to clinical trials that target glutamatergic and GABAergic functions in ASD; however, these studies have failed to yield positive results for primary outcome measures. The GABA-B receptor agonist arbaclofen, for example, did not show significant benefit on its primary outcome measure in a phase 2 clinical trial for ASD (6). The high degree of heterogeneity within ASD likely contributes to these failures, as a treatment designed to target one biological etiology of ASD may not have a beneficial effect on patients with ASD resulting from perturbations in other biological pathways.

## Heterogeneity and biomarkers in ASD

Heterogeneity in ASD can be observed in multiple dimensions, from core symptom pattern to cognitive or communication ability to identifiable risk factors. Genetics has been proposed as a method of subtyping autism (7–17). Rare variants with high penetrance that are directly involved in the etiology of ASD have provided major insights for development of novel therapeutics, while other genes serve as risk factors for ASD that may act in concert with other genetic or environmental risk factors (1, 7–10). However, therapeutics designed to target one specific etiology have an unknown impact on other etiologies of ASD. Additionally, there is a need for greater understanding of pleiotropy within each specific etiology, whereby one might respond to a specific treatment but not another within this group.

In hopes of examining common downstream pathways of the effects of individual etiologies on neural systems, other biomarkers have been assessed in ASD, including markers of brain structure and activity (EEG, imaging) (18–27). A recent study by Ellegood et al. found that 26 different ASD-associated mouse models converged onto three clusters of brain anatomical features from MRI (28). This suggests that neuroimaging may be a powerful tool in the identification of ASD subtypes with specific treatment response, despite genetic heterogeneity; although cost and feasibility issues may limit neuroimaging, particularly in young and more impaired ASD patients. Other types of biomarkers that may be helpful include epigenetic (29, 30), transcriptomic (31–33) (Beversdorf et al.), proteomic (34), and metabolomic markers (35, 36), as well as neurobehavioral measures such as eye-tracking and pupillometry (37–40), actigraphy (41), and psychophysical measures (42). The presence or absence of co-occurring medical (seizures, sleep disturbances, gastrointestinal conditions) and psychiatric conditions (aggression, anxiety, attentional deficits) also contributes to heterogeneity and certainly impacts the approach to treatment.

Heterogeneity is also seen in the core domains of ASD, including social communication and reciprocity deficits, repetitive behaviors/hyperfocused interests, and sensory symptoms. With such disparate symptoms, it may be difficult to formulate ASD severity along a single dimension or to model this unitary diagnosis in epidemiological research or in animal models. The Research Domain Criteria (RDoC) initiative at the National Institute for Mental Health (43) focuses on specific behavioral or cognitive domains within psychiatric or neurodevelopmental diagnoses and may be more tractable for research that spans methods. In support of this, data-driven brain imaging studies have found that brain networks contribute to social communication in a manner that is not diagnosis specific (44). Furthermore, recent studies of the structure of ASD symptoms have suggested four or more distinct social communication dimensions and five separate restricted/repetitive behavior subdomains (45–49). Targeting specific symptom domains would seem advantageous for such a heterogeneous condition as ASD. Recognizing heterogeneity across multiple dimensions, however, it is possible that an intervention may benefit a specific symptom domain in one specific etiology of ASD, with uncertainty about whether this will extend to the broader group of individuals with ASD diagnoses (2).

Within the heterogeneity of ASD, some biomarkers may predict a subpopulation with common disease mechanisms and may therefore be predictive of treatment efficacy. As one of the few examples of the potential utility of biomarkers to dissect heterogeneity within ASD treatment studies, low baseline plasma oxytocin level predicted response to intranasal oxytocin for social responsivity; although this did not replicate in a larger study (50). There are other obvious opportunities to tap into this approach. Alterations in the glutamatergic and GABAergic systems are found with some consistency in ASD postmortem brain studies (51–53), as well as *in vivo* with magnetic resonance spectroscopy (MRS), albeit with some variability across brain regions (54–56). Some large clinical trials for core symptoms of ASD targeted glutamatergic (memantine) and GABAergic (arbaclofen) systems (6). It is possible that direct or indirect markers of GABAergic or glutamatergic system activity, such as MRS (57) (Nair et al.) or EEG gamma band activity (58), would have been valuable in predicting response in a subgroup of individuals, recognizing that no significant effect was seen in the overall group of participants with ASD. Whole blood serotonin (59, 60) or serotonin receptor binding on positron emission tomography (61–65) could similarly predict responses to treatments targeting serotonergic pathways (66). Psychophysical reactivity indicative of sympathetic/parasympathetic tone (67) could identify subjects that may be more responsive to adrenergic treatments (68). In other cases, we may not have obvious biomarker candidates to parse the heterogeneity in ASD treatment studies.

Additionally, the developmental trajectory must be considered in any approach, as mechanisms of actions that impact the developmental trajectory of neural systems at one stage may have an entirely different relevance at a later stage (69). Among the well-replicated imaging findings in ASD is anatomical overgrowth in the first post-natal years (70–74), and some continue to have larger heads later in life resulting from this (75, 76). It would seem that administration of an agent affecting growth trajectories would have remarkably different effects at different ages. Additionally, the impact of the developmental trajectory is likely critical for a wide variety of other factors as well. Thus, temporal factors must also be accounted for in the heterogeneity of ASD to best facilitate individualized treatment approaches and to move toward personalized medicine in ASD.

## Incorporation of a biomarker exploration phase (phase 2m) in clinical trials

The incorporation of rich biobehavioral data to allow subgrouping of participants in clinical trials has the potential to identify which subjects are most likely to respond to a given treatment, and which clinical signs or symptoms are most responsive to that treatment (2, 77, 78). However, the current template of phases for drug development does not regularly incorporate this. In clinical drug development, **phase 1** trials are “dose escalation” or “experimental medicine” trials, focused on the safety and tolerability of drugs, and pharmacokinetics and pharmacodynamics are also assessed. These are followed by **phase 2** trials, where the findings of the first phase are harnessed for further safety, pharmacokinetic, and pharmacodynamic assessment with optimization of dosing and endpoints to be targeted in subsequent phases. **Phase 3** is the confirmatory therapeutic trial, or pivotal trial, conducted in a double blinded manner in a larger population, with statistical power to achieve the predetermined target outcomes based on the phase 2 findings. Successful phase 3 trials are followed by drug approval and marketing, with subsequent **phase 4** studies using observational monitoring to evaluate adverse reactions too infrequent to be detected in phase 3, for monitoring clinical efficacy in the broader population, and to assess cost effectiveness (79). Given the heterogeneity in ASD, it is unreasonable to expect any drug to benefit the majority of individuals, but ASD clinical trials have not had sufficient sample sizes to detect improvement in a subset. While the pharmacodynamic aspect of Phase 1 and 2 trials might be used to identify useful biomarkers and precision medicine targets, this has not commonly been the case for autism drug development. Not surprisingly, then, drug development programs in ASD have typically failed in phase 2 or 3.

**Figure 1:**
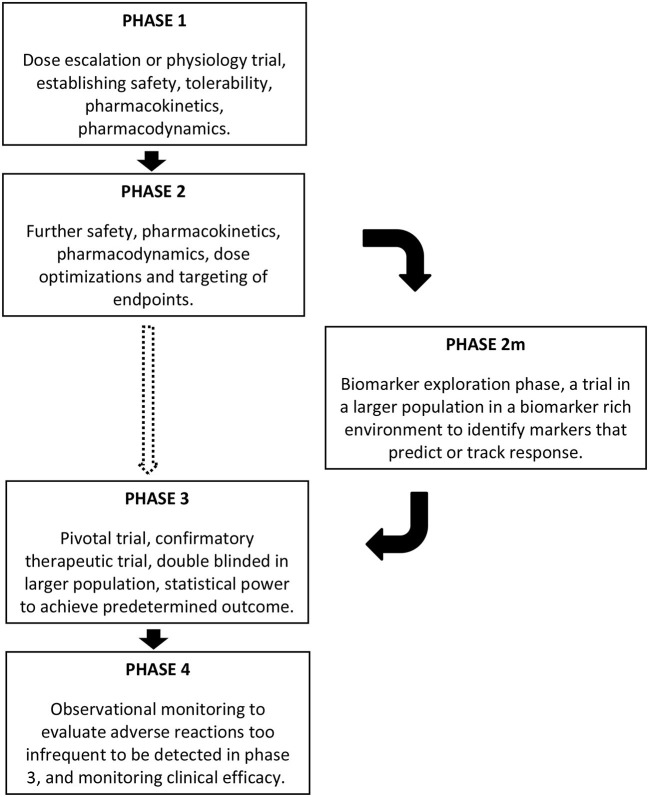
Schematic representing the location of a phase 2m in clinical trials, after phase 1 and phase 2, and before phase 3, if the need for a biomarker exploration phase is warranted based on the nature of the clinical condition. The dashed arrow moving directly from phase 2 to phase 3 might be appropriate for some conditions, but incorporating the phase 2m seems warranted in conditions such as ASD.

A strategy, therefore, must be implemented early in the clinical trial pathway (Figure 1), for identifying biomarkers that can facilitate and inform future trials of the drug in development. In light of the failures of recent large ASD trials (5), we propose that early in Phase 2, a study or studies that could be considered as **phase 2m** (marker exploration phase) should include a rich set of biomarkers that are assessed in a moderately large population of participants to gain an understanding of which subjects respond best, thereby informing the final design and statistical power of later phase 2 and 3 trials. To maximize the richness of the biomarker monitoring, it would be tempting to use a design where all patients will receive the drug, however an open label design is at risk of identifying biomarkers that predict spurious (placebo-related or spurious) response. Blinded crossover designs or staggered start designs might be an appropriate alternative. The participants' developmental stage would also need to be considered as critical marker in this phase. Some markers might be mechanistic, such as biomarkers of GABAergic activity that could predict response to GABAergic agents in ASD. A broader biomarker profiling approach that spans phenotypic subgroups whose mechanistic basis or effects are not fully understood, such as macrocephaly, hyperserotonemia, or elevated IL-6, would better allow the matching of treatments with biomarkers that were not be predicted *a priori*. Other critical questions that could also be addressed include whether earlier intervention could lead to improvement not only in symptoms at the time of the trial but also an improved developmental trajectory. Thus, age of participation and long-term follow-up may be other crucial components to consider for incorporation in future clinical trials.

## Conclusions

With our improving understanding of the genetic and environmental etiologies of ASD and the effects on specific neural systems during distinct developmental epochs, this information can be used for optimization of future clinical trials. By incorporating studies that focus on the predictive value of baseline biomarkers, while also exploring biomarkers that change with treatment and may index response, we can improve the likelihood of success in **phase 3** clinical trials. Integrated approaches to better understand the heterogeneity of autism have been initiated by large collaborations that include clinical trials, such as the Autism Innovative Medicines Study–2-Trials (AIMS-2-Trials) (80–82), as well as the Province of Ontario Neurodevelopmental Disorders (POND) Network (83). Additionally, recent work in the Autism Biomarker Consortium for Clinical Trials (ABC-CT) has been developing neurobehavioral markers, including EEG/ERP, in the hope that they can be used to monitor ASD in clinical trials (84–86). This wealth of data may guide the planning for optimal biomarker choices in the **phase 2m** setting, with mechanistic markers that reflect the function of the neurobiological system(s) targeted by the treatment and other neurobehavioral outcomes that serve as more general indices of ASD symptomatology. Importantly, we will not know which markers will be the best to predict and track response until after the **phase 2m** is completed. The information yielded by this, though, would likely help contribute to improved outcomes for precision medicine optimization in phase 3—and will result in fewer trials that fail to achieve statistical significance despite having a subset of good responders. Furthermore, intervention with an individualized approach at earlier ages is likely to have a larger effect on developmental trajectories. In combination with impactful behavioral therapies (87–91), this approach, implemented early in development, may have an even greater impact on the overall burden of ASD over a lifetime (2, 92).

## Author contributions

DB generated the first draft and every other author made major contributions and agreed on the final text. All authors contributed to the article and approved the submitted version.
